# Superiority of Interferon-Free Regimens for Chronic Hepatitis C: The Effect on Health-Related Quality of Life and Work Productivity

**DOI:** 10.1097/MD.0000000000005914

**Published:** 2017-02-17

**Authors:** Zobair M. Younossi, Maria Stepanova, Rafael Esteban, Ira Jacobson, Stefan Zeuzem, Mark Sulkowski, Linda Henry, Fatema Nader, Rebecca Cable, Mariam Afendy, Sharon Hunt

**Affiliations:** a Department of Medicine, Center for Liver Diseases, Inova Fairfax Hospital, Falls Church, VA; b Betty and Guy Beatty Center for Integrated Research, Inova Health System, Falls Church, VA; c Center for Outcomes Research in Liver Diseases (COR-LD), Washington, DC; d Internal Medicine, Hepatology, and Liver Unit, Universitari Vall D’ Hebron, Barcelona, Spain; e Department of Medicine, Mount Sinai Medical Center, New York, NY; f Department of Internal Medicine, Goethe University Hospital, Frankfurt, Germany; g Department of Medicine, Johns Hopkins University, Baltimore, MD.

**Keywords:** direct-acting antivirals, fatigue, work productivity

## Abstract

Patient-reported outcomes (PROs) such as quality of life and work productivity are important for measuring patient's experience. We assessed PROs during and after treatment of hepatitis C virus (HCV) patients.

Data were obtained from a phase 3 open label study of sofosbuvir and ribavirin (SOF + RBV) with and without interferon (IFN). Patients completed 4 PRO assessment instruments (SF-36, Functional Assessment of Chronic Illness Therapy—Fatigue, Chronic Liver Disease Questionnaire— HCV, Work Productivity and Activity—Specific Health Problem) before, during, and after treatment.

A total of 533 patients with chronic HCV were enrolled; 28.9% treatment-naïve, 23.1% cirrhotic, 219 received IFN + SOF + RBV and 314 received IFN-free SOF + RBV. At baseline, there were no differences in PROs between the IFN-free and IFN-containing treatment arms (all *P* > 0.05). During treatment, patients receiving IFN + SOF + RBV had a substantial impairment in their PROs (up to −24.4% by treatment week 12, up to −8.3% at week 4 post-treatment). The PRO decrements seen in the SOF + RBV arm were smaller in magnitude (up to −7.1% by treatment week 12), and all returned to baseline or improved by post-treatment week 4. By 12 weeks after treatment cessation, patients who achieved sustained viral response-12 showed some improvement of PRO scores regardless of the regimen (up to +7.1%, *P* < 0.0001) or previous treatment experience. In multivariate analysis, the use of IFN was independently associated with lower PROs.

IFN-based regimens have a profoundly negative impact to PROs. By contrast, the impact of RBV on these PROs is relatively modest. Achieving HCV cure is associated with improvement of most of the PRO scores.

## Abstract 2

Supplemental Digital Content is available in the text

## 1 Introduction

The new direct-acting antiviral agents (DAAs) for treatment of hepatitis C virus (HCV) have revolutionized HCV treatment with high sustained viral response (SVR) rates and superior patient-reported outcomes (PROs).^[1–12]^ Although new interferon (IFN)-free and ribavirin (RBV)-free regimens are currently being developed, both IFN and RBV remain a part of certain regimens.^[1–4]^

Previous studies have shown that treatment-naïve HCV patients treated with RBV and IFN experience a significant PRO impairment.^[13,14]^ In particular, the use of IFN causes substantial side effects, including debilitating ones such as severe depression, which, in turn, affect patients’ ability to sustain treatment long enough to obtain a cure whether through medical discontinuation or through patients’ nonadherence. The use of RBV also has been shown to decrease PROs during treatment. However, the PRO data in patients who are retreated after having experienced another course of treatment have not been reported. Therefore, the aim of this study was to evaluate PROs during treatment with and without the use of IFN in patients who participated in a prior study of an IFN-containing or an IFN-free DAA-based regimen and has not achieved SVR.

## 2 Methods

Data were obtained from a phase 3 multicenter open label study investigating the use sofosbuvir (SOF); the study protocol GS-US-334-0109, ClinicalTrials.gov identifier NCT01625338. The target population was patients with all genotypes chronic HCV infection who participated in another study of an SOF-based regimen and did not achieve SVR after receiving active treatment or a placebo; in this study, patients who received a placebo were considered to be treatment-naive. The original intent of this trial was to provide the best opportunity to achieve SVR to participants of prior clinical trials who had experienced a treatment failure or happened to be assigned to a placebo arm. For this study, patients were assigned to receive either IFN + SOF + RBV for 12 weeks or SOF + RBV for 12 or 24 weeks; no randomization or blinding was used. Exclusion criteria were coinfection with HBV or human immunodeficiency virus, pregnancy, history of clinical hepatic decompensation, or the use of immunosuppressants, or other substances as specified in the study report.

We used the medical history collected at screening for the study participants to identify patients with a history of psychiatric disorders, sleep disorders, fatigue or asthenia, and type 2 diabetes or hyperglycemia. Adverse events related to treatment, as confirmed by the study investigators, were grouped into 9 disorder types depending on the organ system involved: blood and lymphatic, gastrointestinal, musculoskeletal and connective tissue, nervous, psychiatric, skin and subcutaneous tissue, fatigue, flu-like symptoms, and other disorders, as described previously.^[11,12]^

### 2.1 Assessment of PROs

In this study, PROs were collected as secondary endpoints using standard instruments.^[15–19]^ All instruments were self-administered by patients prior to initiation of any study-related activities at baseline (day 1) visit, treatment week 12, and at post-treatment weeks 4, 12, and 24 while blinded to their most recent HCV RNA levels.

A Short Form-36 (SF-36) questionnaire is a generic instrument, which has been extensively validated in a variety of populations. It is used to calculate 8 health-related quality of life (HRQL) domains: Physical Functioning, Role Physical, Bodily Pain, General Health, Vitality, Social Functioning, Role Emotional, and Mental Health. The 2 summary scores, which are designed to be linear combinations of these 8 domains, summarize physical and mental health. The 2011 US population norms were used for normalization.^[15]^

The Functional Assessment of Chronic Illness Therapy—Fatigue (FACIT-F) is another widely used HRQL instrument with a fatigue-specific component. It includes a core which consists of the Physical, Emotional, Social, and Functional Well-Being domains and the Fatigue Scale (FS).^[16]^ All domains add up to the total FACIT-F score.

The Chronic Liver Disease Questionnaire—HCV (CLDQ-HCV) is a disease-specific instrument that targets HRQL impairment in patients with chronic HCV infection. It includes 4 HRQL domains: Activity/Energy, Emotional, Worry, and Systemic, which are averaged to the total CLDQ-HCV score.^[17]^

The Work Productivity and Activity—Specific Health Problem (WPAI:SHP) is a different PRO instrument, which is widely used to quantify impairment in patients’ work productivity and daily activities that, as patient believes, is a consequence of a specific health problem (HCV infection, for the purpose of this study). It has 2 domains. The work productivity impairment domain is a sum of impairment in work productivity due to missed work hours (absenteeism) and due to decreased productivity while working (presenteeism); this domain is assessed in employed patients only. The other domain is activity impairment domain, which is impairment in daily activities other than work; it is assessed regardless of employment.^[18]^ Unlike other instruments, all domains of WPAI:SHP are supposed to be inversely related to health status; that is, a greater impairment indicates worse health, and vice versa.

We also calculated SF-6D health utility scores, which are preference-based measures for health necessary for calculation of quality-adjusted years of life in economic analyses; an SF-36 instrument and a nonparametric Bayesian model were used as previously described.^[19]^

### 2.2 Statistical analysis

The treatment regimens were grouped into IFN-free (SOF + RBV) and IFN-containing (IFN + SOF + RBV) regardless of the duration. For all patients, all individual PRO domains, summary PROs, and health utility scores were calculated at each study time point and were further used for calculation of changes (decrements or improvements) in PROs and utilities from patients’ own baseline levels, thus, making patients their own controls. For the purpose of comparison between multiple PROs domains, these domains were transformed from their original scales to a universal 0 to 100 scale with greater scores representing better well-being.

All demographic and clinical parameters, as well as PROs and changes in those, were summarized as mean ± standard deviation or frequency (percentage) in the treatment arms separately, and were compared between the study arms using Wilcoxon rank sum nonparametric test (continuous parameters) or Pearson chi-squared test (categorical parameters). The decrements/improvements in PROs at the study time points from patients’ own baseline were tested for significance using Wilcoxon sign-rank test for matched pairs; a *P* value of 0.05 was used as a threshold for significance. In a separate round of analysis, independent predictors of summary PROs and SF-6D utility scores were assessed at the study time points separately using multiple linear regressions with stepwise selection of predictors; only predictors with *P* < 0.05 were left in the final regression models. In the regression analyses, demographic and clinical variables together with the treatment regimen were tested as potential predictors. All analyses were run in SAS 9.3 (SAS Institute, Cary, NC).

The study was separately approved by each site's Institutional Review Board. Participants signed an informed consent before being enrolled in the trial.

## 3 Results

There were 533 patients in this study (Table 1). Of those, 219 patients received 12 weeks of IFN + SOF + RBV, 114 patients received 12 weeks of SOF + RBV, and 200 patients received 24 weeks of SOF + RBV.

**Table 1:**
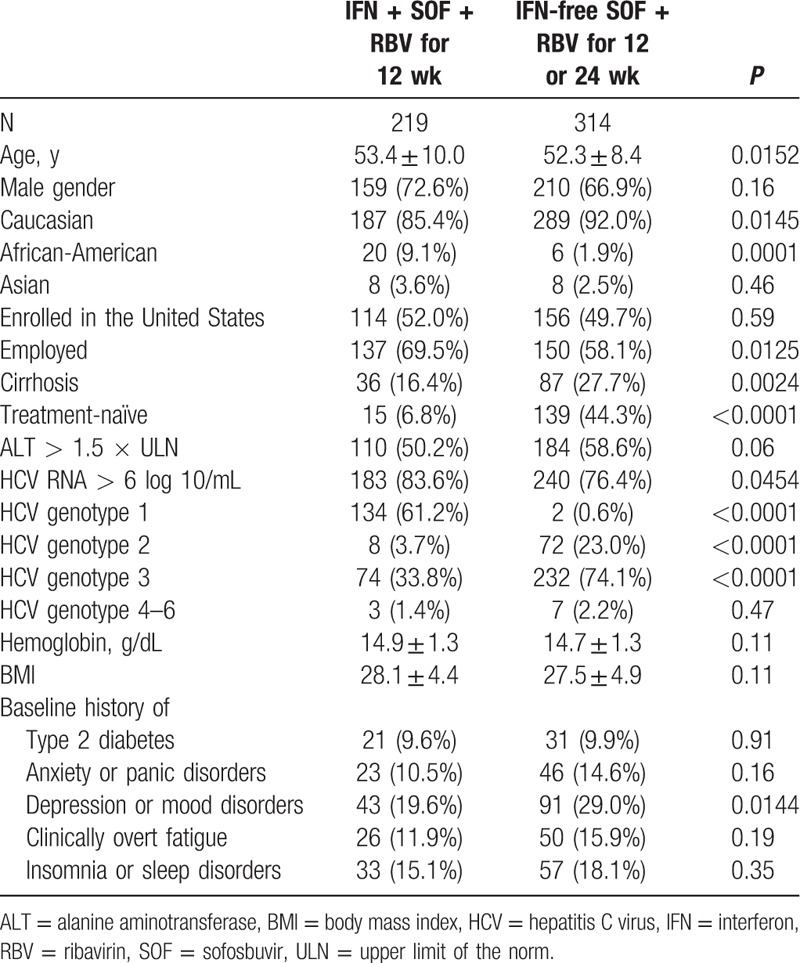
Baseline demographics and clinical presentation of the study cohort.

The demographic and clinical parameters of patients receiving IFN-containing versus IFN-free treatment are listed in Table 1. Patients receiving an IFN-containing regimen were older, more likely African-American, less cirrhotic and treatment-naïve, had predominantly HCV genotype 1, and reported having less depression or mood disorders at screening (all *P* < 0.05, Table 1). On the other hand, patients assigned to an IFN-free SOF + RBV regimen were predominantly HCV genotype 3 or 2 (Table 1).

During treatment, significantly more patients in the IFN + SOF + RBV arm experienced at least 1 treatment-related side effect when compared to the SOF + RBV arm: 89.0% versus 69.7% (*P* < 0.0001). The most frequently reported side effects were fatigue (52.0% vs 30.2%, *P* < 0.0001), depression and other psychiatric issues (45.2% vs 30.6%, *P* = 0.0006), and joint or muscle soreness (39.3% vs 13.1%, *P* < 0.0001) (Table 2). The overall rate of SVR was higher in the IFN + SOF + RBV arm: 82.6% versus 75.5% in SOF + RBV (*P* = 0.048). That included SVR-12 of 79.85% in HCV genotype 1 patients treated with IFN + SOF + RBV, and 90.5% in IFN + RBV + SOF arm versus 69.8% in SOF + RBV arm in HCV genotype 3 patients (*P* = 0.0003).

**Table 2:**
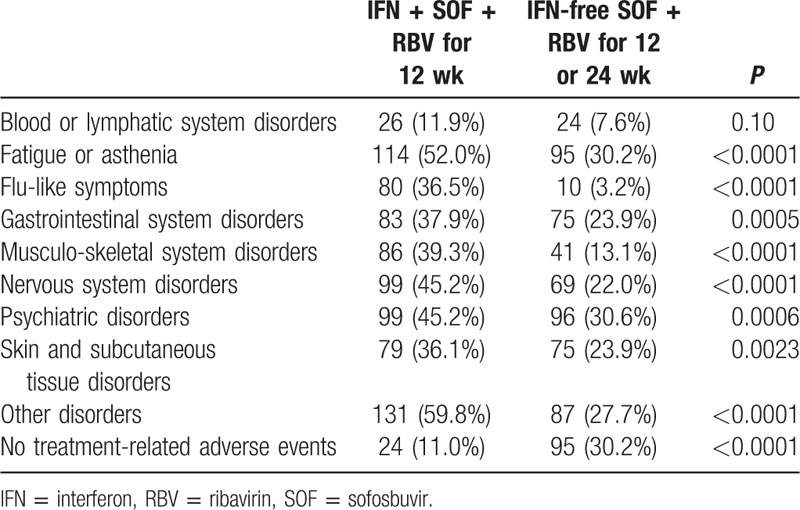
Treatment-related adverse events.

### 3.1 PROs during treatment with and without interferon

At baseline, despite some difference in demographic and clinical presentation, there were no difference in PROs between the 2 treatment arms (all *P* > 0.05) (Supplementary Table 1).

After 4 weeks of treatment with IFN + SOF + RBV, the PRO scores were found to be significantly lower than patients’ own baseline scores (Supplementary Fig. 1). The negative impact of treatment was found to be most apparent in the domains of patients’ Work Productivity (the greatest average decrease of 18.1 on a universal 0–100 scale), Role Physical of SF-36 (average −16.0), Activity/Energy of CLDQ-HCV (−15.9), Activity of WPAI:SHP (−15.5), Physical Well-Being of FACIT-F (−15.3), and FS of FACIT-F (−13.8) (all *P* < 0.0001). The average decrement across 26 studied PROs at that time point was −9.7 points.

**Figure 1:**
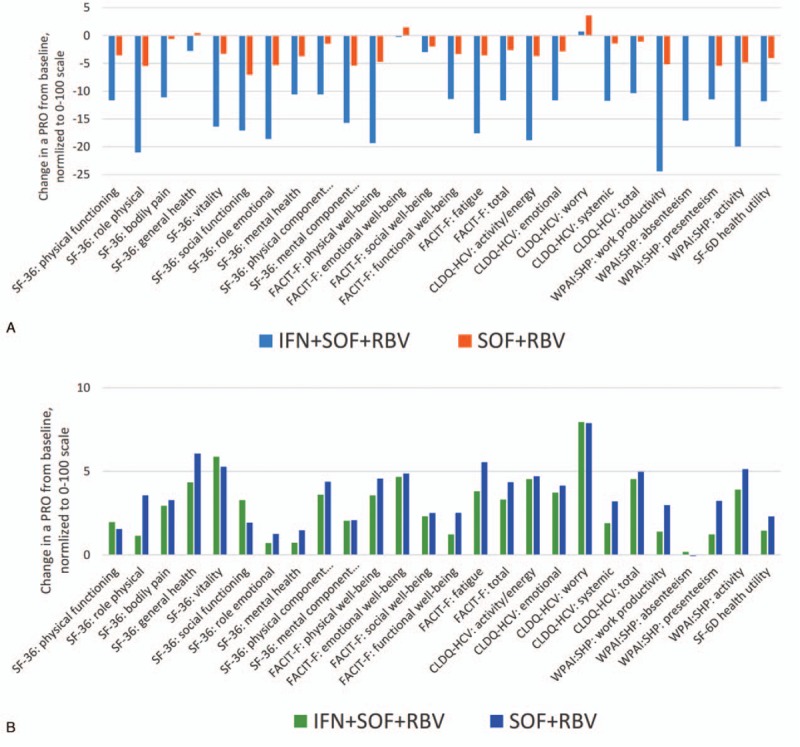
Average changes in PROs from baseline to (A) treatment week 12 (all *P* < 0.05 between regimens except for FACIT-F: emotional well-being and FACIT-F social well-being), and (B) post-treatment week 12 (in patients with SVR-12 only; all *P* > 0.05 between regimens). All PROs were transformed to a universal 0 to 100 scale. FACIT-F = Functional Assessment of Chronic Illness Therapy—Fatigue, PRO = patient-reported outcome, SVR = sustained viral response.

These significant decreases in scores continued throughout treatment with IFN + RBV + SOF (Fig. 1A). Specifically, at 12 weeks, the decrements were up to −24.4 points from baseline; the largest decrements were again observed in Work Productivity and a number of physical functioning-related domains including Role Physical of SF-36, Activity of WPAI:SHP, Physical Well-Being and FS of FACIT-F, and Activity/Energy of CLDQ-HCV (all *P* < 0.0001). The average decrement across 26 PROs was −12.8 points, and the only 2 PRO domains in which no statistically significant decrement was found were the Worry domain of CLDQ-HCV and the Emotional Well-Being domain of FACIT-F (Fig. 1A).

The PRO scores in the IFN + RBV + SOF arm remained low until 4 weeks post-treatment, predominantly in the same areas of impaired Work Productivity (average −8.3), Role Physical (−5.3), Role Emotional (−7.9), Activity (−6.2), and Social Functioning (−5.7) (all *P* < 0.002; the average across PROs −3.3) (Supplementary Fig. 2). Despite this, by post-treatment week 12 in patients with SVR, no PRO remained lower than the baseline level, and 14 out of 26 PRO domains improved relative to baseline (by up to +8.0, and by +4.2 on average; all *P* < 0.05) (Fig. 1B). Additionally, by week 24 after treatment discontinuation, all but 4 average PRO scores (Physical Functioning, Bodily Pain, Social Functioning of SF-36, and Absenteeism of WPAI:SHP) were significantly above their baseline levels (all *P* < 0.05).

**Figure 2:**
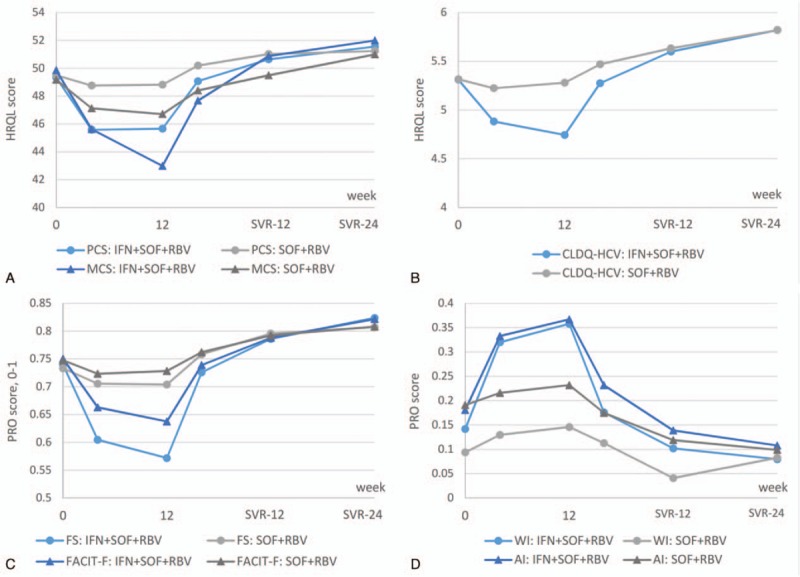
Summary PROs during and after treatment with IFN + RBV + SOF and IFN-free RBV + SOF. AI = activity impairment, FS = Fatigue Scale, IFN = interferon, MCS = Mental Summary Score, PCS = Physical Summary Score, PRO = patient-reported outcome, RBV = ribavirin, SOF = sofosbuvir, WI = work productivity impairment. All *P* < 0.05 between SOF+RBV and IFN+SOF+RBV treatment arms at treatment weeks 4 and 12.

In contrast to the IFN-containing regimen, the on-treatment PRO decrements in patients receiving an SOF + RBV-based treatment were smaller in magnitude (Fig. 2). In particular, the average PRO decrement by treatment week 4 was −2.0, with the greatest decrements observed in Social Functioning of SF-36 (−4.4), Physical Well-Being of FACIT-F (−4.0), Role Emotional of SF-36 (−3.8), and Presenteeism of WPAI:SHP (−3.5) (all *P* < 0.005) (Supplementary Fig. 1). Similarly, the decrements observed by treatment week 12 were also substantially smaller in comparison to those seen in the IFN-containing arm: up to −7.1 points in Social Functioning of SF-36 and −2.9 points across all studied PROs (Fig. 1A).

Furthermore, in patients who received an IFN-free SOF + RBV regimen, all PROs returned to baseline levels or started to improve by post-treatment week 4 (improvements up to +4.1 in the Worry domain of CLDQ-HCV, average improvement across 7 improved PROs +2.1; all *P* < 0.05); no residual PRO decrement relative to baseline was observed at that time point (Supplementary Fig. 2). By 12 weeks after treatment cessation, in patients with SVR-12, all but 6 PRO scores significantly improved in patients treated with SOF + RBV by up to +7.1 points (+4.2 on average; all *P* < 0.03); note that these improvements are now similar to those observed in the IFN-containing arm (all *P* > 0.05) (Fig. 1B), and a similar observation was made at post-treatment week 24 as well.

In multivariate analysis, after adjustment for demographic and clinical predictors of PROs, which were similar to those reported previously^[8–12,20–23]^ and included site location, history of psychiatric disorders, fatigue, cirrhosis, the use of IFN in addition to SOF + RBV was independently associated with a greater impairment in PROs during and soon after treatment discontinuation (Table 3). In particular, the magnitude of association (beta) ranged from −8.1 to −22.2 at treatment week 12 (all *P* < 0.0001) and from −4.6 to −11.9 at post-treatment week 4 (all *P* < 0.02). By post-treatment week 12, the Physical Component Summary of SF-36, the CLDQ-HCV score, and Work Productivity Impairment score were still associated with treatment received: from −4.5 to −5.6 points for the use of IFN in comparison to the reference SOF + RBV regimen (all *P* < 0.05).

**Table 3:**
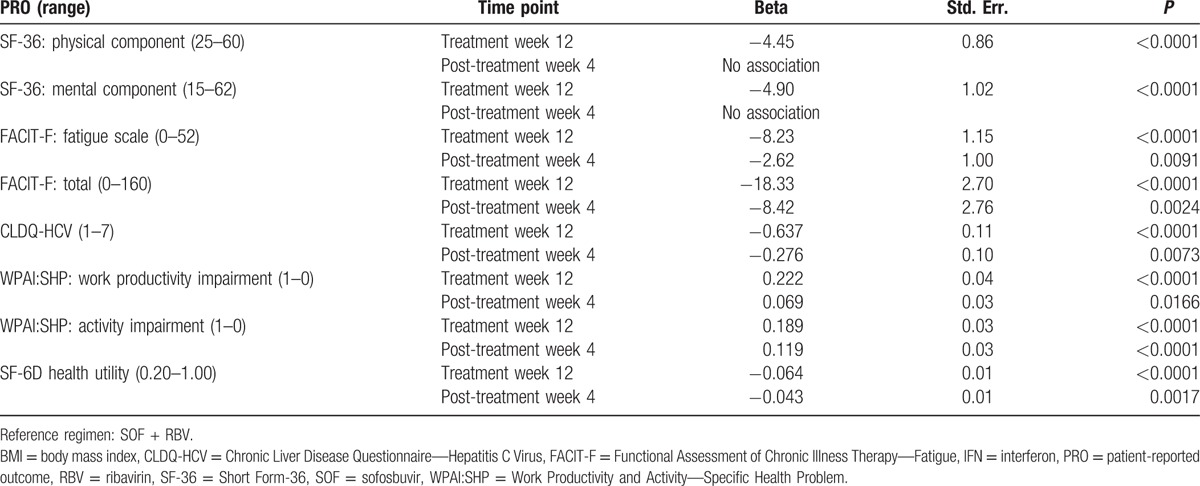
Independent association of the use of an IFN-containing treatment regimen with PROs during and soon after treatment cessation (adjusted for age, gender, location, BMI, history of psychiatric disorders, fatigue, and treatment history).

### 3.2 Subgroup analysis: PROs in treatment-experienced patients treated with IFN

In a separate round of analysis, we tested the hypothesis that the effect of IFN on PROs may be less pronounced in treatment-experienced patients who have already been treated with IFN. For that purpose, we used the subgroup of treatment-experienced patients currently treated with IFN + SOF + RBV, and compared baseline PROs and treatment-emergent changes in PROs between those who had received an IFN-containing regimen before and those who had received an IFN-free regimen with or without RBV.

As shown in Supplementary Table 2, most of the baseline PROs in patients with history of IFN treatment were significantly higher when compared to baseline PROs in patients who were being retreated after an IFN-free regimen. These PROs included 4 domains of SF-36 (Role Physical, General Health, Social Functioning, and Role Emotional), all but one domains of FACIT-F, all domains of CLDQ-HCV, Activity Impairment of WPAI:SHP, and the SF-6D health utility score (*P* < 0.05); nearly all of these PROs remained higher during treatment with IFN in this study. However, no difference in treatment-emergent changes in PROs with reference to patients’ own baseline levels was found between patients treated with IFN after having failed an IFN-free versus IFN-containing regimen neither in univariate nor in multivariate analysis (all *P* > 0.05).

## 4 Discussion

The purpose of this study was to investigate the impact of alternative hepatitis C treatment regimens on PROs. In this study, we have found that patients who were treated with a regimen containing IFN experienced a substantial decrease in their PRO scores during and even shortly after treatment. According to the multivariate analysis, the use of IFN was also found to be an independent predictor of substantial PRO impairment, and that association was observed up to 12 weeks after treatment discontinuation. Although PROs were also impacted by IFN-free regimens that contained RBV, the magnitude of such impact is much smaller. These data are consistent with previously published PRO data.^[2–10,21–23]^

Across both IFN-free and IFN-containing treatment arms, PRO domains that were most affected by the treatment were the domains primarily associated with daily functioning and physical activity. Thus, it is imperative for healthcare practitioners to assist patients in dealing with these areas by identifying strategies patients can pursue to stay active and social.

A number of previous studies of the use of IFN for treatment of chronic HCV infection have shown the negative effect of IFN on PROs.^[13,14]^ In this study, we have shown that the impact of IFN-containing and IFN-free RBV-containing regimens on PROs of patients who are retreated is similar to those who have never been treated or have been treated with and IFN-free regimen only. In fact, experience with previous IFN-based treatment does not seem to predispose patients for better or worse experience during another course of treatment. Although HCV patients who were treatment-experienced with IFN had higher baseline PRO scores, most of these were likely due to previously applied strict treatment eligibility criteria.

A major limitation of this study is related to the original clinical trial design, which lacks both randomization and blinding. We, however, believe that the bias caused by this could be adequately accounted for by multivariate analysis.

In conclusion, treatment-experienced patients who were retreated with an IFN-containing regimen experienced significantly more impairment of their PROs in comparison to patients who were treated with an IFN-free regimen regardless of duration. However, it is important to note that, albeit accompanied by an unfavorable patients’ well-being profile, the IFN-containing regimen was still associated with a higher chance of SVR, especially in patients with HCV genotype 3. These 2 outcomes represent a trade-off that patients who have failed a DAA-based regimen may face, although the use of IFN, hopefully, will be revisited once new DAAs come to market and provide this cohort of patients with more treatment options.

## Supplementary Material

Supplemental Digital Content
