# Supplementary material for: Superiority of Interferon-Free Regimens for Chronic Hepatitis C: The Effect on Health-Related Quality of Life and Work Productivity
Source: Medicine (Baltimore). 2017 Feb 17;96(7):e5914. doi: 10.1097/MD.0000000000005914 (PMC5319496; doi:10.1097/MD.0000000000005914)
Supplement: Supplemental Digital Content [file medi-96-e5914-s001.doc]

**Supplementary Figure 1**. Average changes in PROs from baseline to treatment week 4; all p<0.05 between the regimens except for Emotional and Social Well-Being of FACIT-F.


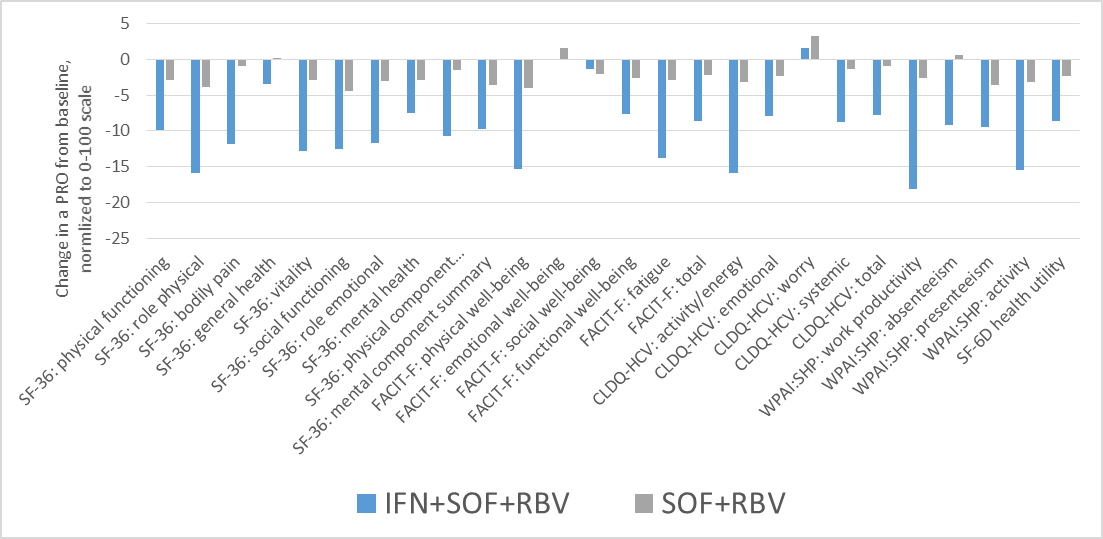


**Supplementary Figure 2**. Average changes in PROs from baseline to post-treatment week 4.


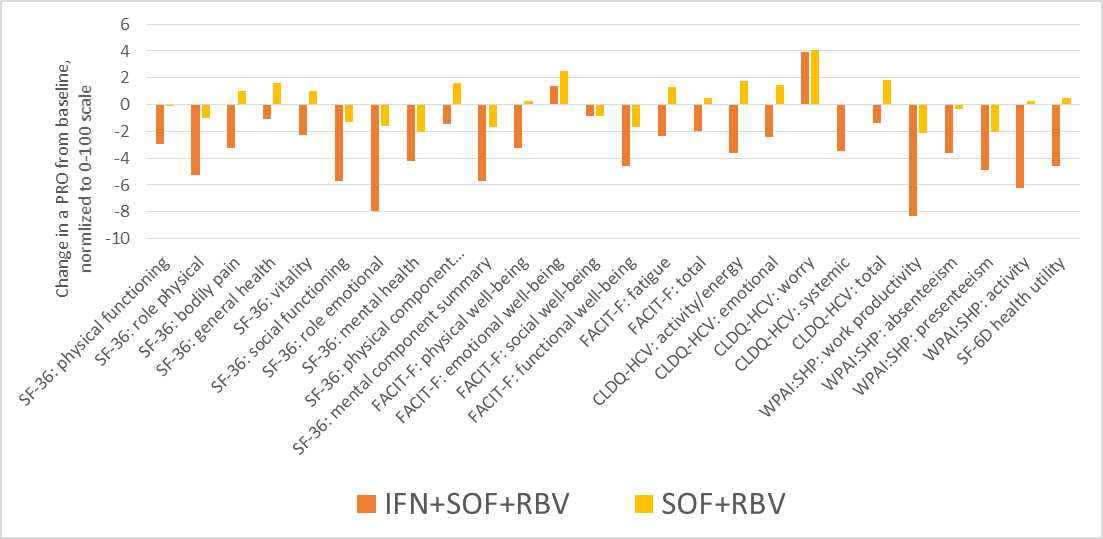


**Supplementary Table 1**. Baseline patient-reported outcomes in the study cohort.

| PRO (range) | IFN+SOF+RBV | IFN-free SOF+RBV | P |
| --- | --- | --- | --- |
| SF-36: physical functioning (0-100) | 79.7 ± 23.2 | 80.0 ± 22.8 | 0.70 |
| SF-36: role physical (0-100) | 76.2 ± 26.8 | 75.4 ± 27.0 | 0.60 |
| SF-36: bodily pain (0-100) | 71.2 ± 25.8 | 71.2 ± 25.5 | 0.98 |
| SF-36: general health (0-100) | 62.3 ± 22.8 | 59.9 ± 23.2 | 0.25 |
| SF-36: vitality (0-100) | 58.9 ± 23.5 | 59.0 ± 23.5 | 0.95 |
| SF-36: social functioning (0-100) | 80.0 ± 24.7 | 78.7 ± 25.4 | 0.60 |
| SF-36: role emotional (0-100) | 82.8 ± 23.4 | 80.4 ± 24.8 | 0.24 |
| SF-36: mental health (0-100) | 74.0 ± 18.5 | 72.8 ± 19.7 | 0.61 |
| SF-36: physical component summary (25-60) | 49.3 ± 9.2 | 49.5 ± 9.0 | 0.93 |
| SF-36: mental component summary (15-62) | 49.9 ± 10.1 | 49.2 ± 10.7 | 0.40 |
| FACIT-F: physical well-being (0-28) | 22.6 ± 5.1 | 22.8 ± 5.3 | 0.25 |
| FACIT-F: emotional well-being (0-24) | 18.2 ± 4.4 | 18.4 ± 4.3 | 0.71 |
| FACIT-F: social well-being (0-28) | 21.1 ± 5.8 | 20.7 ± 6.5 | 0.90 |
| FACIT-F: functional well-being (0-28) | 19.9 ± 5.9 | 19.5 ± 6.6 | 0.75 |
| FACIT-F: fatigue (0-52) | 38.4 ± 11.7 | 38.1 ± 11.9 | 0.86 |
| FACIT-F: total (0-160) | 120.0 ± 26.6 | 119.6 ± 29.7 | 0.75 |
| CLDQ-HCV: activity/energy (1-7) | 5.29 ± 1.36 | 5.24 ± 1.40 | 0.88 |
| CLDQ-HCV: emotional (1-7) | 5.44 ± 1.17 | 5.36 ± 1.26 | 0.75 |
| CLDQ-HCV: worry (1-7) | 5.44 ± 1.21 | 5.58 ± 1.22 | 0.15 |
| CLDQ-HCV: systemic (1-7) | 5.08 ± 1.21 | 5.09 ± 1.29 | 0.87 |
| CLDQ-HCV: total (1-7) | 5.31 ± 1.10 | 5.32 ± 1.17 | 0.71 |
| WPAI:SHP: work productivity (1-0) | 0.142 ± 0.242 | 0.094 ± 0.186 | 0.28 |
| WPAI:SHP: absenteeism (1-0) | 0.040 ± 0.136 | 0.030 ± 0.130 | 0.06 |
| WPAI:SHP: presenteeism (1-0) | 0.101 ± 0.175 | 0.064 ± 0.119 | 0.28 |
| WPAI:SHP: activity (1-0) | 0.181 ± 0.258 | 0.191 ± 0.256 | 0.60 |
| SF-6D health utility (0.20-1.00) | 0.705 ± 0.148 | 0.705 ± 0.147 | 0.85 |

**Supplementary Table 2**. Baseline patient-reported outcomes in treatment-experienced patients treated with IFN.

| PRO (range) | Previously treated  with IFN | Previously treated  w/o IFN | p |
| --- | --- | --- | --- |
| SF-36: physical functioning (0-100) | 81.29 ± 22.90 | 80.08 ± 21.88 | 0.49 |
| SF-36: role physical (0-100) | 81.74 ± 24.56 | 72.03 ± 27.12 | 0.0034 |
| SF-36: bodily pain (0-100) | 74.95 ± 22.94 | 69.86 ± 27.30 | 0.30 |
| SF-36: general health (0-100) | 69.05 ± 20.69 | 58.09 ± 22.59 | 0.0004 |
| SF-36: vitality (0-100) | 61.08 ± 22.56 | 58.40 ± 23.08 | 0.27 |
| SF-36: social functioning (0-100) | 85.80 ± 22.55 | 76.39 ± 23.34 | 0.0004 |
| SF-36: role emotional (0-100) | 87.46 ± 20.90 | 79.21 ± 24.17 | 0.0010 |
| SF-36: mental health (0-100) | 76.74 ± 17.40 | 72.47 ± 18.26 | 0.07 |
| SF-36: physical component summary (25-60) | 50.94 ± 8.16 | 48.60 ± 9.59 | 0.06 |
| SF-36: mental component summary (15-62) | 51.62 ± 9.34 | 48.70 ± 9.84 | 0.0300 |
| FACIT-F: physical well-being (0-28) | 23.59 ± 4.23 | 22.05 ± 5.07 | 0.05 |
| FACIT-F: emotional well-being (0-24) | 18.89 ± 4.02 | 17.79 ± 4.36 | 0.0414 |
| FACIT-F: social well-being (0-28) | 21.43 ± 6.16 | 20.67 ± 5.72 | 0.21 |
| FACIT-F: functional well-being (0-28) | 20.95 ± 6.02 | 19.47 ± 5.15 | 0.0340 |
| FACIT-F: fatigue (0-52) | 40.89 ± 10.06 | 37.06 ± 11.76 | 0.0252 |
| FACIT-F: total (0-160) | 125.52 ± 24.93 | 117.04 ± 25.15 | 0.0135 |
| CLDQ-HCV: activity/energy (1-7) | 5.56 ± 1.20 | 5.15 ± 1.33 | 0.0237 |
| CLDQ-HCV: emotional (1-7) | 5.70 ± 1.04 | 5.25 ± 1.13 | 0.0022 |
| CLDQ-HCV: worry (1-7) | 5.71 ± 1.12 | 5.25 ± 1.17 | 0.0034 |
| CLDQ-HCV: systemic (1-7) | 5.33 ± 1.10 | 4.96 ± 1.20 | 0.0311 |
| CLDQ-HCV: total (1-7) | 5.57 ± 0.98 | 5.15 ± 1.04 | 0.0016 |
| WPAI:SHP: work productivity (1-0) | 0.13 ± 0.24 | 0.17 ± 0.25 | 0.34 |
| WPAI:SHP: absenteeism (1-0) | 0.04 ± 0.15 | 0.04 ± 0.12 | 0.41 |
| WPAI:SHP: presenteeism (1-0) | 0.09 ± 0.17 | 0.12 ± 0.18 | 0.24 |
| WPAI:SHP: activity (1-0) | 0.13 ± 0.22 | 0.21 ± 0.27 | 0.0136 |
| SF-6D health utility (0.20-1.00) | 0.73 ± 0.14 | 0.69 ± 0.15 | 0.0188 |
